# Overweight and Undernutrition in the Cases of School-Going Adolescents in Wolaita Sodo Town, Southern Ethiopia: Cross-Sectional Study

**DOI:** 10.1155/2018/8678561

**Published:** 2018-03-27

**Authors:** Dereje Yohannes Teferi, Gudina Egata Atomssa, Tefera Chane Mekonnen

**Affiliations:** ^1^ABH Service PLC, Addis Ababa, Ethiopia; ^2^College of Medicine and Health Sciences, Haramaya University, Dire Dawa, Ethiopia; ^3^Department of Public Health, College of Medicine and Health Sciences, Wollo University, Dessie, Ethiopia

## Abstract

**Background:**

This study aimed to assess the prevalence of malnutrition and associated factors among school adolescents in Wolaita Sodo town, Southern Ethiopia.

**Methods:**

A school-based cross-sectional study was conducted from May 18–June 10, 2015. A multistage sampling was used to select a random sample of 690 adolescents from selected schools. Data on sociodemographic information were collected by using an interviewer-administered questionnaire, and anthropometric measurements were made by using a digital Seca scale and height measuring board by trained data collectors. Data were entered into Epi-Data version 3.1 software and exported to SPSS version 20. World Health Organization (WHO) Anthro-plus software was used to analyze anthropometric data. Both binary and multinomial logistic regression analyses were done to identify factors associated with the malnutrition of adolescents.

**Result:**

The overall prevalence of thinness, stunting, and overweight/obesity among school adolescents was 4.7% (95% CI: 3%–6.4%); 5.2% (95% CI: 3.4%–7%); and 5.0% (95% CI: 3.4%–6.7%), respectively. Being male (AOR = 4.07; 95% CI: 2.35–7.02), learning at a government school (AOR = 0.37; 95% CI: 0.20–0.65), mothers with no formal education (AOR = 4.03; 95% CI: 1.82–8.92), owning no cattle (AOR = 4.92; 95% CI: 2.08–11.64), skipping meals (AOR = 1.70; 95% CI: 1.05–2.74), and illness in 2 weeks prior to survey (AOR = 2.67; 95% CI: 1.49–4.78) were significantly associated with thinness. However, males, students who had their house, and no cattle were more likely to develop overweight/obesity. Maternal education of secondary school (AOR = 0.214; 95% CI: 0.054–0.846) was significantly associated with the stunting.

**Conclusion:**

The study showed the coexistence of undernutrition and overnutrition among school adolescents in the study area. There needs to implement evidence-based school nutrition education and health policies and programs to improve nutritional status of adolescents and timely taking action to limit obesity-related health problems.

## 1. Background

World Health Organization defines adolescence as a period of transition between childhood and adulthood (10–19 years) and as a period where most important physiological changes occurred. They constitute about one billion (18%) of the world's population [1, 2].

Even though prime causes of adolescent nutritional problem in the developing world are due to inadequacies of quality and quantity of food, both of the extremes of the nutrition spectrum are linked with a range of adverse health conditions. Overweight and obese adolescents are at risk factors of developing noncommunicable diseases and also suffer from psychological problems such as stigmatization and poor self-esteem [2–5].

Obesity is the risk factor for a range of conditions, including cardiovascular disease, diabetes mellitus, arthritis, and some cancers [6–8].

Adolescence serves as a window of opportunity to break intergenerational cycle of malnutrition and to build a nutritionally healthy adult life. Some nutritional problems originating early in life can be potentially corrected, unless it will lead to life threatening noncommunicable diseases in adulthood. It is the actual period to shape and consolidate healthy eating and lifestyle behaviors, thereby preventing or postponing the onset of nutrition-related chronic disease in adulthood [9].

Information regarding nutritional status of adolescents in Ethiopia is not well documented, and they were the neglected segment of the population [10]. The national prevalence of overweight and/or obesity among adolescents is not found. However, individual study from Addis Ababa, Ethiopia, showed that adolescent overweight/obesity is an emerging challenge and it was about 8% and revealed that private school-going adolescents were more affected [8]. Studies confirmed that nearly three out of four obese adolescents remain obese as adults, increasing their risk of heart disease, type 2 diabetes, stroke, and cancers [11, 12].

But still their feeding problems and nutritional status determination using appropriate anthropometric measurements are not enough investigated in these age groups [9, 13]. Adolescents are suffering from chronic noncommunicable diseases that have been mostly neglected by the international community: diagnosing and treating NCDs and other chronic conditions during adolescence need to be incorporated into national programmes [14].

The available studies focused on factors related to reproductive issues rather than factors associated with normal growth and development of adolescents [15]. Therefore, studies that indicate the actual level of adolescents' nutritional problem and investigate related factors have greater potential to design appropriate intervention and are an alarming for timely taking action for noncommunicable disease prevention.

## 2. Methods and Materials

### 2.1. Study Design and Area

A school-based cross-sectional study was carried out from May 18–June 10, 2015, in Wolaita Sodo town. The Wolaita Sodo town is located at 385 kilometers from south west Addis Ababa and 160 kilometers west of regional town, Hawassa, at 6°49′ N latitude and 39°47′ E longitude and at an altitude of about 1900 m. Sodo town is the capital town of the Wolaita zone, one of the 13 zones in Southern Ethiopia, with an area of 830 square kilometers. According to the 2007 Ethiopian National Population and Housing Census, the population of the town is projected to be about 137,522 for July 2014, with male to female ratio 1.03 : 1 [10]. The town was structured into 3 subcities and 11 kebeles. There are 10 secondary and 20 primary schools. Of the schools in the town, 16 were governmental and 14 were private schools. None of them had school feeding program or other funding opportunity. The main staple food of the population is teff and maize.

### 2.2. Population and Eligibility Criteria

All primary, secondary, and preparatory school adolescent students were considered as a source population, whereas all regular students in the selected schools aged between 10 and 19 years where the study population, and all randomly selected students aged between 10 and 19 years in selected schools were constituted the sampling population.

School-going adolescents aged between 10 and 19 years who had been attending regular program were included in the study. However, adolescents who had spinal curvature (unable to stand erectly), pregnant, and edema due to some other pathological causes were excluded.

### 2.3. Sample Size Determination and Sampling Procedure

The sample size was calculated for the three outcomes (thinness, stunting, and overweight/obesity), and the maximum was taken. The sample size was determined by using a single population proportion formula by considering the following assumptions: 95% confidence level, 16.7% the prevalence of overweight and obesity among school adolescents in North West, Ethiopia [16], margin of error 4%, design effect 2.0, and 5% nonresponse rate. Finally, 690 school adolescents were taken as the sample size.

A multistage sampling technique was used in this study. Among the total of 27,601 students in the town, 21,405 students were enrolled in government schools and 6,196 students were enrolled in private schools. The schools were first stratified in two: governmental and nongovernmental schools. Using the lottery method four schools from each stratum were selected. In the selected schools, one section per grade was selected randomly that makes a total of 25 sections. The total sample size was proportionally allocated to the number of students found in the 25 sections. The allocated sample size per each section was also reallocated to the number of female and male students. Simple random sampling was used, by using ENA for SMART software to select the study subjects randomly.

### 2.4. Study Variables

Nutritional status among school adolescents in the forms of thinness, normal, and overweight was the prime measure of interest in addition to measuring the chronic irreversible growth faltering (stunting) among adolescents. Data on sociodemographic variables (such as age, gender, family size, educational status of household head, occupational status of father, occupational status of mother, type of school, and family's residence), socioeconomic status, eating habit/nutritional factors, adolescent's behavioral factors (physical work, alcohol drinking, smoking, and eating disorders), environmental factors (presence or absence of latrine, source of drinking water, and personal hygiene), reproductive health (history of pregnancy and age of menarche), and history of medical illness in the past two weeks were deeply investigated. We defined *thinness*: BMI-for-age *Z*-score less than −2 standard deviation from new WHO 2007 reference population; *stunting*: height-for-age *Z*-score less than −2 standard deviation of the new WHO 2007 reference population; *overweight*: BMI-for-age *Z*-score between +1 and +2 standard deviation for new WHO 2007 reference population; and *obesity*: BMI-for-age *Z*-score right to +2 standard deviations for the new WHO 2007 reference population.

### 2.5. Data Collection

A structured interviewer-administered questionnaire was used to collect data. The questionnaire was developed based on the conceptual framework after thorough review of different literature, and it covered a range of information on socioeconomic and demographic characteristics, adolescents' dietary practice, reproductive health, lifestyle, and sanitation of adolescents. A height measuring board and portable digital Seca scale were used for measurement of height and weight, respectively. A total of twelve data collectors comprising ten diploma holder nurses, with previous experience of data collection, and two BSc holder supervisors were employed to collect the data. The questionnaire was developed from preestablished known sources like reports of an expert committee by WHO and other literatures conducted in developing countries including Ethiopia [2, 6, 10].

### 2.6. Anthropometric Measurements

The weight was measured using a well-calibrated, portable digital Seca scale. Students were weighted standing on the scale with their shoes off. The digital Seca scale was a self-zeroing scale and was calibrated each day before starting data collection by putting 2 kg iron bars on each scale to ascertain accuracy. The students were measured by taking off shoes, heavy clothes, and mobile from the pocket and by standing upright on the foot mark on a scale according to WHO recommendations [17]. Height was measured using the portable studiometer, which consisted of an anthropometry with a simple triangular headboard. In taking height, the students were made to stand straight with their shoes off and head held erect such that the external auditory meatus and the lower border of the eye were in one horizontal plane (Frankfurt plane). The buttocks, shoulder blades, and heel touched the scale with knees and legs together, and arms hanging naturally by the sides. A movable triangular headboard was brought against the crown of the head, and height measurement was read off at maximum inspiration. The height was measured to the nearest 0.1 cm and weight to the nearest 0.1 kg [17]. All measurements were taken three times, and the average was taken. Anthropometric measurements were converted to height-for-age and BMI-for-age *Z*-scores and compared to the new 2007 WHO reference data for 5–19 years, using WHO Anthro-plus computer program [18]. Adolescents with BMI-for-age *Z*-score (BAZ) <−5 or >5 were considered as outliers.

### 2.7. Data Quality Control and Standardization

To assure the quality of data, training was given to the data collector and supervisors for two days prior to data collection. The training covered the proper filling of the questionnaire, the use of the weight and height scales, and the proper taking of height and weight in order to minimize inter- and intraobserver errors. To assure the accuracy of anthropometric measurement, standardization test was done in 10 adolescents before the actual survey and systematized based on the result. The technical error of measurement (TEM) was found to be in the acceptable range. Two days before the actual study began, the questionnaire was pretested and validated in 5% of adolescents (not included in the sample) selected from different schools and some modifications were made based on response categories. To improve the quality of the data, the data collectors were closely supervised, and each completed questionnaire was checked to ascertain all questions were properly filled and corrected by the principal investigator. To minimize interobserver variability, during pretesting the coefficient of variation was checked and it was 1.8%, and nature of the data was explored thoroughly to check influential outliers and missed values during analysis.

### 2.8. Data Processing and Analysis

First, data were checked for completeness and consistency in data entry and cleaning. Then, the data were entered into the Epi-Data version 3.1 and exported to SPSS version 20. Anthropometric data and other essential variables were exported to WHO Anthro-plus software, a computer program which converts anthropometric data into *Z*-scores of the indices, BAZ and HAZ, taking age and sex into consideration using WHO 2007 population references.

Descriptive statistics using frequency, proportions, mean, standard deviation, and cross tabulations were used to present the study results. Even though multinomial logistic regression does not make any assumptions of normality, linearity, and homogeneity of variance for the independent variables, maximum likelihood estimation, model fitness, classification accuracy, and presence of multi-colinearity were explored before performing the final model. Normality of all continuous variables was checked by using the Kolmogorov–Smirnov test at *p* value > 0.05. Binary logistic regression was done to determine the associations between each independent variable and outcome variable: stunting. Multinomial logistic regression was performed to investigate factors associated with adolescent nutritional status (thinness, normal, and overweight). Odds ratio with 95% confidence intervals was used to see the strength of association between different variables. A *p* value less than 0.05 was considered as statistically significant.

## 3. Result

### 3.1. Sociodemographic Characteristics of Participants

A total of 655 school-going adolescents participated in the study, with the response rate of 95.2%. Of the study subjects, 340 (51.9%) were males and more than half (377 (57.6%)) were from government schools. Regarding the age of the respondents, the majority (532 (81.2%)) were 15–19 years old and the median age of the respondents was 17 years with interquartile range (IQR) being 3 years. As to the family of the respondents, more than half (397 (60.6%)) of the respondents were from large family members (>5 members). Regarding the education level of the respondent's father, about 274 (41.80%) were college and university completed. About half (333 (50.8%)) of the respondents' fathers were government employees (Table 1).

### 3.2. Dietary Habits and Food Frequency of the Students and Their Families

The student's dietary habit showed that most (471 (71.9%)) of the respondents consumed teff as a staple food. Among the families of the respondents, 482 (73.6%) consumed food from market purchases and 88 (13.5%) consumed from their own product and market purchase. A majority (621 (94.8%)) of the respondents consumed fruit in a week of which 207 (31.6%) consumed fruit three days a week. Likewise, 382 (59.1%) of the respondents were taking meat, and 251 (55.6%) had a practice of eating once a week. On the other side, 583 (89%) of the respondents confirmed that they consumed foods containing pulse legumes and lentils (Table 2).

Concerning meal frequency and dietary habit, more than half (404 (61.7%)) of the respondents consumed regular meal three times a day. Regarding skipping regular meal, 304 (46.4%) of the respondents skipped at least one or two of a regular meal. More than half of the respondents (414 (63.2%)) claimed that they did not feel hunger due to a shortage of food in a month. Majority (611 (93.3%)) of the respondents never received any vitamin or mineral supplementation. None of the schools had a school feeding program.

### 3.3. Adolescents' Characteristics on Lifestyle, Health, and Sanitation

More than three-fourth (574 (87.6%)) of the respondents did not engage in work besides education. Almost the entire respondents (653 (99.7%)) did not smoke cigarettes while 623 (95.1%) of adolescents had never ever drunk alcohol.

Regarding the source of water, the majority (618 (94.4%)) of the respondents used water from safe sources (protected well, spring, and tap). Almost all of the respondents' family (99.8%) had latrine of which 575 (87.8%) had dry pit latrine and 79 (12.1%) had flush toilets. More than three-fourth (572 (87.3%)) of the respondents used soap to wash hands after the toilet. The majority (575 (87.8%)) of respondents had no attack of illness within two weeks prior to the survey. Some of the common illnesses were abdominal pain (23 (3.5%)), malaria (15 (2.3%)), typhoid fever (9 (1.4%)), and upper respiratory disease (9 (1.4%)). Around 40 (6.1%) reported that they have difficulty with seeing at night, and 56 (8.5%) have neck swelling (goiter) (Table 3).

Among female respondents, about 267 (84.8%) claimed that they start menstruating. The median and mean ages of menarche were 14 years and 13.69 years, respectively. Only 0.6% of female adolescents had previous history of pregnancy.

### 3.4. Nutritional Status of Adolescents

The mean height and weight were 162.43 cm and 51.96 kg, respectively. The mean BAZ and HAZ were −0.58 and −0.49, respectively. Among all school-going adolescents, 8 (1.2%) were severely thin (<−3 SD), 24 (3.6%) were thin (=−3 SD and <−2 SD), 159 (90.2%) were within the normal range (=−2 SD), 32 (4.9%) were overweight (+1 SD and +2 SD), and 2 (0.3%) were obese (=+2 SD).

**Figure 1:**
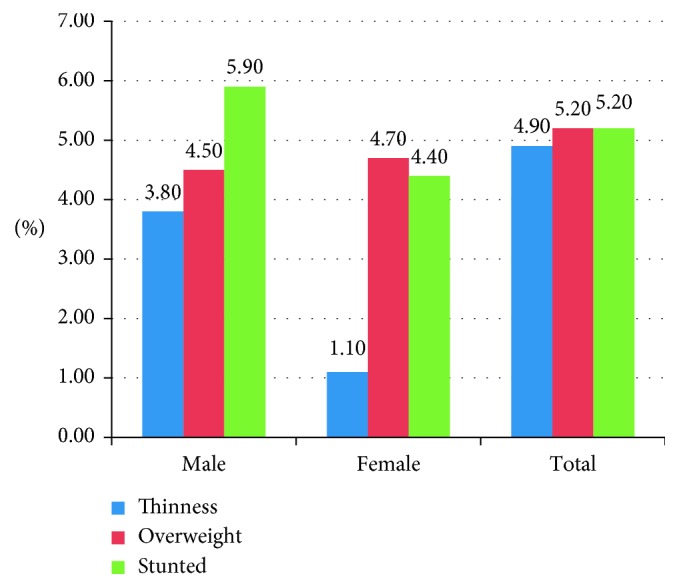
Distribution of nutritional status by sex of respondents among school-going adolescents in primary, secondary, and preparatory school of Wolaita Sodo town, May 2015.

The overall prevalence of thinness (BAZ < −2 SD) and stunting (HAZ <−2 SD) of school adolescents was 4.8% (95% CI: 3%–6.7%) and 5.2% (95% CI: 3.4%–7%), respectively. Thinness and stunting were more prevalent among males than females: 7.4% versus 1.9% and 5.9% versus 4.4%, respectively. The prevalence of overweight and obesity is 4.8% and 0.3%, respectively. The prevalence of overweight/obesity was significantly higher among girls than boys, 9.5% versus 0.9% (Figure 1).

The nutritional status of school-going adolescents was compared to the current new WHO 2007 reference population of age 5–19 years' curve. The BMI-for-age of female adolescents is nearly normally distributed, whereas males are negatively skewed which shows that the mean BMI-for-age of male adolescents was lower than the new WHO 2007 reference populations.

### 3.5. Factors Associated with Malnutrition of School Adolescents

From the result of multiple logistic regression analysis, students from government schools, male adolescents, mothers with no formal education, being male as household head, students from rented houses, having no intake of fruits per week, having no cattle in the house, skipping meals, and having illness in the last two weeks of the survey showed statistically significant association with thinness. Similarly, male adolescents, school adolescents having their own house, and students who have no cattle and have one to three cattle revealed statistical association with overweight. The odds of thinness among male school adolescents were nearly four times higher than the odds of thinness among girls (AOR = 4.07; 95% CI: 2.35–7.02), but they were less affected by overweight (AOR = 0.1; 95% CI: 0.03–0.16) than female adolescents in reference to normal weight school adolescents. School-going adolescents whose mothers had no formal education were also four times more likely to have chronic energy deficiency (thinness) (AOR = 4.03; 95% CI: 1.82–8.92) but had no association with overweight (AOR = 0.38; 95% CI: 0.13–1.11) as compared to school adolescents from mothers having college and university educational level. The odds of being thin (AOR = 1.70; 95% CI: 1.05–2.74) among school adolescents who had missed their meals were two times higher as compared to the odds of thinness among students who did not skip their meals.

However, skipping meals had no relation to the occurrence of overweight (Table 4).

In addition to the above, the study tried to investigate influencing factors of stunting (HAZ < −2 SD) using binary logistic regression. Even though some variables were identified as associated factors for stunting, after controlling for confounding effect of variables in the final step, only mothers' education had associations with stunting. Adolescents whose mothers had secondary education were 78.6% more likely to stunting as compared to adolescents whose mothers had college and university education (Table 5).

## 4. Discussion

This study revealed that the overall prevalence malnutrition (thinness, overweight, and stunting) was 4.8% (95% CI: 3%–6.4%), 4.8% (95% CI: 3.2%–6.6%), and 5.2% (95% CI: 3.4%–7%), respectively. The prevalence of both overweight and obesity was 5.0% (95% CI: 3.4%–6.7%). The prevalence of undernutrition was more or less comparable to the study conducted in Addis Ababa; the prevalence of thinness and stunting was 7.2% and 6.2% [8]. The report also goes in line with the nutritional status reported from Turkish adolescents where the rate of being underweight and stunted was 5.0% and 4.4% [19]. The prevalence of thinness is much lower than those reported from the Mekelle city (37.8%) [16], Ambo town (27.2%) [20], Chiro town (24.4%) [21], Malaysia (16%) [22], and Addis Ababa (13%) [23]. Similarly, the study has shown a lower prevalence of thinness than International Center for Research on Women (ICRW) reports from different African countries, such as Senegal (29.8%), Benin (23%), and Sudan (25%) [22]; West Bengal (42.4%) [24]; and Nigeria (18.9%) [25]. The difference is mainly due to the difference in socioeconomic backgrounds in the study areas. When boys were compared to girls, there was a higher prevalence of thinness among boys than girls (7.4% versus 1.9%). This might be due to more variation in maturation time in boys than girls, for which girls attained maturation earlier than boys. The difference can also be explained that, in our community, boys are more engaged in hard physical work and other out-door activities than girls that they have higher energy expenditure compared to girls. The differences in prevalence of thinness by sex are also demonstrated by the study conducted in Ambo town (29.8% versus 24.6%) [20], Chiro town (34.2% versus 10.4%) [21], Addis Ababa (9.9% versus 2.6%) [8], Wardha of South India (75% versus 25%) [26], and West Bengal (52.1% versus 32%) [24]. Manyanga et al. reported from large cross-sectional data from Global School-based Student Health Survey (GSHS) conducted in seven African countries, based on WHO 2007 growth chart, that males had a higher prevalence of being thin in every country [6]. By contrast, Mondal and Terangpi from Assam, North India, reported that prevalence of thinness was slightly higher among girls (14.9%) than boys (12.05%) [27].

Regarding the sex specific prevalence of stunting, there is higher prevalence of stunting among boys (5.9%) than girls (4.4%). This study also agrees with reports from 16 demographic and health survey from Sub-Saharan Africa [28]. However, Nitish Mondal and his colleague pointed that, in Assam, North India, prevalence of stunting was similar among boys (50.1%) and girls (50.2%) [27]. There is a higher prevalence of stunting among late adolescents (5.6%) than early adolescents (3.3%). By contrast, study done in Chiro town showed there is a high prevalence of stunting in early adolescents than late adolescents (7.8% versus 6.8%) [21].

About overweight and obesity, the prevalence of overweight and obesity is 4.7% and 0.3%, respectively. Yetubie et al. [20] also reported that 4.4% of adolescents were overweight in Ambo town. This is higher than overweight/obesity reported from Mekele city (2.4%) [16], Chiro town, West Hararge [21], and rural Wardha, India (2.0%) [6]. This might be due to socioeconomic differences. However, it is lower than those reported from Hawassa city (12.9%) [29] and Addis Ababa (8.5%) [8]. This can be explained by the fact that Hawassa and Addis Ababa are more urban than Wolaita Sodo, and adolescents living there may have a higher chance of feeding modern canned foods and fried junk foods.

The prevalence of overweight/obesity was nearly the same with the prevalence of thinness (5.0% versus 4.7%), indicating both forms of malnutrition coexist in the study area. The prevalence of overweight/obesity in this study tells us that the problem is rapidly increasing as compared to 2011 report of national demographic survey of Ethiopia and large numbers of adolescents are prone for development of noncommunicable diseases. To date, there is no clear and comprehensive intervention strategy for such population segments to reduce overthreatening effect of NCDs in later life. The present study showed that there is a significantly higher rate of overweight/obesity among girls than boys; 9.5% versus 0.9%. This can be explained by the fact that girls attain maturity earlier than boys. It can also be due to, in Ethiopia, female adolescents did household chores such as cooking giving them more access to food. This finding is consistent with the finding of a study conducted in Ambo town [20].

This study revealed that being male, private school, mothers' education, having private house, having more cattle, and had an illness in the last two weeks preceding survey are associated with thinness. Male adolescents were 4 times more likely to be thinner than their counterparts. This can be explained by that boys are maybe engaged in more strenuous activity than girls. This study agrees with a study from Mekelle city and Chiro town where boys are more likely to be thinner than girls, respectively [16, 21]. This also goes in line with the study conducted in African countries which confirms that boys were more affected by thinness than girls due to that boys are encouraged to be autonomous at younger age than girls, meaning that they are more likely to be exposed to infection which leads to nutritional problems [6, 30].

It is expected that adolescents at private schools are relatively from wealthy families, and thus, they might have low prevalence of undernutrition and higher prevalence of overnutrition as compared to their counterparts. However, in contrast to this, the study showed that adolescents learning in private schools were more likely to be thin as compared to those from government schools. This might be due to differences in the feeding habit.

Adolescents from families that do not have cattle were four times more thinner than their counterparts. Cattle are an important source of milk and meat. Families that do not raise their own cattle must purchase milk and meat from the sources outside of the home. Without money, this approach is not likely to be successful and people substitute grain for milk and meat. Yetubie et al. also reported similar findings from Ambo town [20]. The study also revealed that adolescents from families renting their house were more likely to be thin compared to adolescents from families who owned the house. This might be due to unstable lifestyle and low socioeconomic condition among the former ones.

Evidence suggests that the educational status of the family, particularly the mother, was an important determinant of nutritional status of their children [16]. This study also revealed that adolescents whose mothers have no formal education were more likely to be thinner than adolescents whose mothers have college and university education. In contradiction to the above, students of less educated mothers had significantly lower odds for stunting. This may be due to the fact that mothers with low education level will have enough time for caring their children, but mothers with advanced education will be employed by different sectors and thus will not have adequate time. This might be due to the fact that the higher the maternal education, the more the household income increasing the purchasing power of the household. Another possible explanation could be mothers who have attained higher education level might have good awareness about nutrition and has decision making power over the household food choice and might have also good caring capacity of their children. Educated mothers are also more likely to use modern health care [31].

Regarding the health status, 12.2% of adolescents reported that they had an illness in the last two weeks preceding the survey. Adolescents who had an illness within two weeks preceding survey were 3 times more prone to thinness than those who had not. The disease affects nutritional status by reducing appetite and at the same time by increasing nutrient demand of the body. The study done in Jimma [32] and Gujarat, India, [33] showed that adolescents who have poor access to health care are more likely undernourished.

Even though extensive effort was made to enhance the quality of the study, there may be minimal effect on the power of the study due to small sample size. There might be some interobserver variability by the data collector, but standardization and adjustment of scales and reliability of the tool were done regularly.

## 5. Conclusion and Recommendation

The present study successfully documented undernutrition, in terms of thinness and stunting and overweight/obesity among school-going adolescents of Wolaita Sodo town. The prevalence of undernutrition is slightly similar as that of overnutrition, indicating that both forms of malnutrition coexist. There is an increasing trend in the level of overweight/obesity. Being male, mother with no formal education, owning no cattle, and illness in last two weeks preceding survey was associated with being thin. The higher level of maternal education also reduces the rate of stunting among their children. There was a significant difference on the prevalence of adolescent malnutrition for age and sex of adolescent with respect to different nutritional indicators.

## Figures and Tables

**Table 1:** Sociodemographic and economic characteristics of respondents among school adolescents in Wolaita Sodo town, May 2015.

Variable (*N*=655)	Level	Frequency	Percent
School type	Government	377	57.6
	Private	278	42.4

Age (years)	10–14	123	18.8
	15–19	532	81.2

Sex	Male	340	51.9
	Female	315	48.1

Religion	Orthodox	208	31.8
	Protestant	426	65.0
	Muslim	13	2.0
	Catholic	8	1.2

Ethnicity	Wolaita	588	89.8
	Amhara	22	3.4
	Gurage	14	2.1
	Gamo	9	1.4
	Others	22	3.3

Original residence	Urban	519	79.2
	Rural	136	20.8

Residential house	Rent from private	62	9.4
	Owned	550	84.0
	Rent from government	43	6.6

Fathers' education	No formal education	40	6.1
	Grade 1–4	24	3.7
	Grade 5–8	111	16.9
	Grade 9-10	120	18.3
	Grade 11-12	86	13.1
	College and university	274	41.8

Mothers' education	No formal education	71	10.8
	Grade 1–4	62	9.5
	Grade 5–8	181	27.6
	Grade 9-10	141	21.5
	Grade 11-12	59	9.0
	College and university	141	21.5

Family size	≤5	258	39.4
	>5	397	60.6

Monthly income (ETB)	<1000	175	26.7
	1001–2000	126	19.2
	2001–3000	109	16.6
	3001–4000	74	11.3
	>4001	171	26.1

**Table 2:** Dietary habit (food frequency) of respondents among school adolescents of Wolaita Sodo town, May 2015.

Variable (*N*=655)	Level	Frequency	Percent
Type of staple food	Teff	471	71.9
	Maize	181	27.6
	Others	3	0.5

Source of food	Own product	86	13.1
	Market purchased	481	73.4
	Own product/purchase	88	13.5

Meal frequency per day	Twice	26	4.0
	Three times	404	61.7
	Four times	225	34.4

Fruit per week	Yes	621	94.8
	No	34	5.2

Fruit per day/week (*n*=621)	1 day/wk	64	10.3
	2 days/wk	169	27.2
	3 days/wk	207	31.6
	4 days/wk	181	29.1

Fruit per day (*n*=621)	Once	390	59.5
	Twice	174	26.6
	Three times	57	6.9

Pulse and legume	Yes	583	89.0
	No	72	11

Vegetable per week	Yes	611	93.3
	No	44	6.7

Episode of hunger in past 30 days	No hunger	414	63.2
	Once	70	10.4
	Twice	75	11.5
	Three times	40	6.1
	Four times	56	8.5

**Table 3:** Characteristics on behavior, lifestyle, health, and sanitation among school adolescents in Wolaita Sodo town, May 2015.

Variables (*N*=655)	Level	Frequency	Percent
Work besides education	Yes	81	12.4
	No	574	87.6

Smoke cigarette	Yes	2	0.3
	No	653	99.7

Ever drink alcohol	Yes	32	4.9
	No	623	95.1

Latrine type (*n*=654)	Dry pit	575	87.9
	Flush toilet	79	12.1

Water source	Safe	618	94.4
	Unsafe	37	5.6

Cattle in the same house	Yes	186	28.4
	No	469	71.6

Brush teeth per day	No	53	8.1
	At least once	602	91.9

Hand wash before feeding	Not at all	11	1.7
	Sometimes	56	8.5
	Usually	588	89.8

Menses (*n*=315)	Yes	267	84.8
	No	48	15.2

Illness in the last two weeks	Yes	80	12.2
	No	575	87.8

Any chronic illness	Yes	10	1.5
	No	645	98.5

Difficulty in seeing at night	Yes	40	6.1
	No	615	93.9

Neck swelling	Yes	56	8.5
	No	599	91.5

**Table 4:** Factors associated with thinness and overweight of school adolescents, Wolaita Sodo town, South Ethiopia, May 2015.

Nutritional status	Explanatory variables (*N*=655)	*N* (%)	COR (95% CI)	AOR (95% CI)	*p* value
Thinness	*Sex of students*				
	Male	25 (78.1)	3.17 (1.35–7.44)	**4.07 (2.35–7.02)**	0.001
	Female	7 (21.9)			
	*School type*				
	Government	11 (34.4)	0.36 (0.17–0.77)	**0.37 (0.20–0.65)**	0.001
	Private	21 (65.6)			
	*Maternal education*				
	No formal education	6 (18.8)	1.32 (0.44–3.85)	**4.03 (1.82–8.92)**	0.001
	Primary education	10 (31.2)	0.62 (0.24–1.57)	1.08 (0.58–2.02)	0.79
	Secondary education	7 (21.9)	0.53 (0.19–1.46)	0.79 (0.42–1.52)	0.49
	College and university	9 (28.1)			
	*Sex of household head*				
	Male	31 (96.9)	4.11 (0.55–30.61)	**4.66 (1.30–16.69)**	0.01
	Female	1 (3.1)			
	*House ownership*				
	Rented	7 (21.9)	1.86 (0.34–10.12)	**4.11 (1.34–12.59)**	0.01
	Owned	25 (78.1)			
	*Fruit intake per week*				
	No intake	3 (9.4)	2.86 (0.67–12.09)	**3.09 (1.22–7.80)**	0.01
	One day/week	3 (9.4)	1.43 (0.34–5.90)	1.15 (0.46–2.85)	0.75
	Two days/week	11 (34.3)	2.03 (0.73–5.62)	1.92 (0.99–3.69)	0.05
	Three days/week	9 (28.1)	1.34 (0.46–3.82)	1.49 (0.76–2.94)	0.23
	Four days and above	6 (18.8)			
	*Cattle ownership*				
	No cattle	25 (78.1)	3.25 (0.96–10.94)	**4.92 (2.08–11.64)**	0.002
	1–3 cattle	4 (12.5)	2.28 (0.49–10.4)	2.25 (0.84–5.99)	0.1
	Four and above	3 (9.4)			
	*Illness in the past 2 weeks*				
	Yes	7 (21.9)	2.14 (0.89–5.14)	**2.67 (1.49–4.78)**	0.001
	No	25 (78.1)			
	*Work besides education*				
	Yes	2 (6.3)	0.44 (0.10–1.89)	0.45 (0.17–1.17)	0.10
	No	30 (93.7)			
	*Skip meal*				
	Yes	19 (59.4)	1.73 (0.84–5.58)	**1.70 (1.05–2.74)**	0.02
	No	13 (40.6)			

Overweight	*Sex of students*				
	Male	3 (8.8)	0.1 (0.01-0.28)	**0.1 (0.03-0.16**)	0.001
	Female	31 (91.2)			
	*School type*				
	Government	19 (55.9)	0.88 (0.44–1.77)	0.96 (0.57–1.61)	0.89
	Private	15 (44.1)			
	*Maternal education*				
	No formal education	2 (6.1)	0.43 (0.09–2.08)	0.38 (0.13–1.11)	0.07
	Primary education	12 (36.4)	0.74 (0.30–1.82)	0.52 (0.27–1.00)	0.05
	Secondary education	10 (30.3)	0.75 (0.29–1.92)	0.72 (0.39–1.34)	0.31
	College and university	9 (27.2)			
	*Sex of household head*				
	Male	32 (94.1)	2.12 (0.49–9.05)	2.31 (0.92–5.79)	0.07
	Female	2 (5.9)			
	*House ownership*				
	Owned	25 (73.6)	0.63 (0.18–2.19)	**0.37 (0.16–0.86)**	0.01
	Rented	9 (26.4)			
	*Fruit intake per week*				
	No intake	2 (5.9)	1.27 (0.26–6.19)	1.86 (0.66–5.25)	0.23
	One day/week	3 (8.8)	0.95 (0.25–3.64)	1.85 (0.76–4.53)	0.17
	Two days/week	8 (23.5)	0.98 (0.37–2.61)	1.46 (0.76–2.80)	0.25
	Three days/week	12 (35.3)	1.19 (0.49–2.89)	1.63 (0.90–2.96)	0.10
	Four days and above	9 (26.5)			
	*Cattle ownership*				
	No cattle	21 (61.8)	0.81 (0.37–1.76)	**0.37 (0.20–0.69)**	0.002
	1–3 cattle	3 (8.8)	0.51 (0.13–1.91)	**0.36 (0.14–0.89)**	0.02
	Four and above	10 (29.4)			
	*Illness in the past 2 weeks*				
	Yes	5 (14.7)	1.32 (0.49–3.52)	1.15 (0.57–2.31)	0.69
	No	29 (85.3)			
	*Work besides education*				
	Yes	2 (5.9)	0.41 (0.09–1.76)	0.35 (0.10–1.24)	0.10
	No	32 (94.1)			
	*Skip meal*				
	Yes	16 (47.1)	1.05 (0.52–2.11)	1.09 (0.69–1.73)	0.7
	No	18 (52.9)			

The reference category is normal weight adolescents; the bold indicates statistically significant variables; COR, crude odds ratio; AOR, adjusted odds ratio.

**Table 5:** Factors associated with stunting of school adolescents, Wolaita Sodo town, Southern Ethiopia, May 2015.

Variable (*N*=655)	Nutritional status		COR (95% CI)	AOR (95% CI)
	Stunted	Normal		
*Original residence*				
Urban	22 (4.2%)	497 (95.8%)	1	
Rural	12 (8.8%)	124 (91.2%)	2.19 (1.05–4.54)^∗^	2.619 (0.83–8.27)
*Cattle in the same house*				
Yes	17 (9.1%)	169 (90.9%)	2.67 (1.33–5.36)^∗^	2.11 (0.86–5.20)
No	17 (3.6%)	452 (96.4%)	1	
*Source of drinking water*				
Safe source	29 (4.7%)	589 (95.3%)	1	
Not safe source	5 (13.5%)	32 (86.5%)	3.17 (1.15–8.74)^∗^	2.03 (0.66–6.25)
*Fathers' occupation*				
Farmer	8 (7.5%)	99 (92.5%)	1.41 (0.59–3.35)	0.32 (0.09–1.23)
Merchant	3 (2.1%)	143 (97.9%)	0.36 (0.11–1.27)	0.25 (0.61–1.02)
Day laborer	5 (7.2%)	64 (92.8%)	1.36 (0.49–3.82)	0.98 (0.28–3.36)
Government/NGO employee	18 (5.4%)	315 (94.6%)	1	
*Mothers' education*				
No formal education	6 (8.5%)	65 (91.55)	1.21 (0.42–3.47)	0.49 (0.10–2.40)
Primary education	14 (5.8%)	229 (94.2%)	0.801 (0.35–1.85)	0.53 (0.15–1.89)
Secondary education	4 (2.0%)	196 (98.0%)	0.26 (0.08–0.87)^∗^	0.21 (0.05–0.85)^∗^
College and university	10 (7.1%)	131 (92.9%)	1	
*Family size*				
≤5 members	17 (6.6%)	241 (93.4%)	0.63 (0.32–1.27)	2.03 (0.94–4.36)
>5 members	17 (4.3%)	380 (95.7%)	1	
*House ownership*				
Owned	31 (5.6%)	519 (94.4%)	1	
Rent from private	1 (1.6%)	61 (98.4%)	0.27 (0.04–2.05)	1.80 (0.37–8.66)
Rent from government	2 (4.7%)	41 (95.3%)	0.82 (0.19–3.53)	0.31 (0.02–3.97)

COR, crude odds ratio; AOR, adjusted odds ratio; ^∗^statistical significant variable at *p* value < 0.01.

## Data Availability

The datasets during and/or analyzed during the current study are available from the corresponding author on reasonable request.

## Abbreviations

BAZ: BMI-for-age *Z*-score
BMI: Body mass index
CI: Confidence interval
HAS: Height-for-age *Z*-score
SD: Standard deviation.
